# The impact of the school-to-work transition on self-rated health & subjective well-being in Germany

**DOI:** 10.1093/eurpub/ckac129.583

**Published:** 2022-10-25

**Authors:** M Reuter, M Herke, M Richter, K Diehl, S Hoffmann, CR Pischke, N Dragano

**Affiliations:** 1 Heinrich Heine University Düsseldorf, Institute of Medical Sociology, Düsseldorf, Germany; 2 Martin Luther University Halle-Wittenberg, Institute of Medical Sociology, Halle, Germany; 3 Friedrich-Alexander-University, Department of Medical Informatics, Biometry and Epidemiology, Erlangen, Germany; 4 Heidelberg University, Center for Preventive Medicine and Digital Health, Mannheim, Germany; 5 Brandenburg University of Technology, Department of Public Health, Senftenberg, Germany; 6 Technical University of Munich, Department of Sport and Health Sciences, Munich, Germany

## Abstract

**Background:**

During the school-to-work transition (STWT), young people enter different states as vocational training, university or unemployment that may have immediate or long-term effects on health. Since research has not paid much attention to this, we investigate the development of self-rated health (SRH) and subjective well-being (SWB) during the STWT.

**Methods:**

We used data from Starting Cohort 4 of the German National Educational Panel Study (NEPS), a nationally representative cohort of 11,098 ninth graders (50.5% girls) followed over nine years. Linear panel regression analysis with fixed-effects (FE) was used to explore intra-individual changes in SRH and SWB when moving between different STWT states (school, prevocational program, vocational training, university, employment, unemployment, inactivity). FE impact functions were used to compare trajectories of SRH and SWB by states reached after school-leave. Time-varying control variables were age, household composition, and residential area.

**Results:**

School-leave was linked to increases in SRH and SWB, whereas no impact was found for job entry after vocational training or university. Upward transitions (e.g. from a prevocational program to vocational training, from vocational training to university or from unemployment to employment) increased SRH or SWB, while downward transitions (e.g. from vocational training or employment to unemployment) were related to decreases. Over the years after school-leave, we found a decline in SRH and SWB, which was faster in case of transitions to unemployment or prevocational programs directly after school.

**Conclusions:**

Findings suggest that a smooth STWT is key for good health in youth and adulthood. Health and labour market intervention programs should focus on the time after school-leave, especially on those who are not able to find an academic or vocational training position.

